# Manchester Triage System: main flowcharts, discriminators and outcomes of a pediatric emergency care**^1^**

**DOI:** 10.1590/1518-8345.1078.2779

**Published:** 2016-08-29

**Authors:** Camila Amthauer, Maria Luzia Chollopetz da Cunha

**Affiliations:** 2 RN, MSc.; 3 PhD, Associate Professor, Escola de Enfermagem, Universidade Federal do Rio Grande do Sul, Porto Alegre, RS, Brazil.

**Keywords:** Triage, Pediatrics, Emergency Nursing, Pediatric Nursing, Emergency Service, Hospital.

## Abstract

**Objetive:**

to characterize the care services performed through risk rating by the Manchester Triage System, identifying demographics (age, gender), main flowcharts, discriminators and outcomes in pediatric emergency

**Method:**

cross-sectional quantitative study. Data on risk classification were obtained through a search of computerized registration data from medical records of patients treated in the pediatric emergency within one year. Descriptive statistics with absolute and relative frequencies was used for the analysis.

**Results:**

10,921 visits were conducted in the pediatric emergency, mostly male (54.4%), aged between 29 days and two years (44.5%). There was a prevalence of the urgent risk category (43.6%). The main flowchart used in the care was worried parents (22.4%) and the most prevalent discriminator was recent event (15.3%). The hospitalization outcome occurred in 10.4% of care performed in the pediatric emergency, however 61.8% of care needed to stay under observation and / or being under the health team care in the pediatric emergency.

**Conclusion:**

worried parents was the main flowchart used and recent events the most prevalent discriminator, comprising the hospitalization outcomes and permanency in observation in the pediatric emergency before discharge from the hospital.

## Introduction

The emergency service can be regarded as the more complex health care in Brazil, with a demand for service far greater than its absorption capacity. With this increasing demand, it is visible the imbalance between supply and demand for care in these services, making fundamental the reorganization of the work process.^1^. Moreover, the action must be joint to a regulatory system with reference and counter reference, focused on outpatient care and classification within the hospital environment, providing follow-up sites after treatment in the emergency department^2^.

The reception of the patient in the emergency service must be performed by a risk classification protocol, aiming to prioritize the care according to the severity of the case. Among the existing risk classification protocols, the Manchester Triage System (MTS) created in England, stands out as it determines the level of urgency of each patient. The nurse responsible for the classification selects the most appropriate flow chart for the main complaint, medical history and signs and symptoms presented by the patient, a discriminator is found and the patient is classified into one of five categories: Immediate (red), immediate evaluation by a physician; Very Urgent (orange), evaluation within 10 minutes; Urgent (yellow), 60 minutes; Standard (green), 2 hours; and Non-Urgent (blue), 4 hours^3^ ^-^ ^4^.

The nurse is the professional assigned to the evaluation and classification of the patient's risk, guided by a classification protocol, proposing the use of a service flowchart to motivate this professional to reflect on their work process in the context of attention to emergency, replacing the exclusionary screening by a welcoming classification model^5^.

In emergency situations, the child is usually the main victim, as they require special attention on their health characteristics, needing specialized resources for their emergency care^6^. Regarding the risk rating in a pediatric emergency department, it is perceived the need for an organized and systematized health care process to the child's health, plus a structured classification instrument that allows nurses to assess carefully the main complaints of the patient at the time of classification, in order to provide care and proper referral to the suffering, risk and / or harm to the child's health ^7^.

The MTS provides a list of 52 flowcharts, 49 suitable for children. Studies conducted in the Netherlands evaluated the validity of the MTS in a pediatric emergency care, aiming to carry out a detailed assessment of specific patient categories. The studies showed that the MTS has moderate sensitivity and specificity in pediatric emergency care, possibly due to lack of organization of specific flowcharts for pediatric patients^8^ ^-^ ^10^.

In reviewing the literature, there are no studies on the profile of pediatric patients classified with this protocol; so little research has been reported with the MTS analysis in pediatric emergency. It appears that the nurse responsible for the risk rating is often the same that cares for adults and children, without having a more focused look at this age group, considering the particularities that pediatric patients have. Considering the importance of characterizing the care of pediatric patients classified with the MTS the following research question arises: "What are the characteristics of the services performed by the risk rating of the Manchester Triage System in a pediatric emergency of a university hospital?" This study aims at understanding the use of this classification protocol in pediatric patients and may, in the future, justify the development of researches in order to optimize the risk rating from the MTS in the pediatric population. Therefore, this study aimed to characterize the care provided by risk rating through the Manchester Triage System, identifying demographics (age, gender), main flowcharts, discriminators and outcomes in pediatric emergency.

## Method

A cross-sectional quantitative study in a pediatric emergency, linked to an emergency service, located at a university hospital in southern Brazil, which covers the care of adults and pediatric patients through the Brazilian National Health System. With capacity to hospitalize 49 adult patients and nine pediatric patients, this facility serves more than 5,000 patients each month. Of this total, the pediatric emergency serves an average of 1,000 patients per month. From the year 2012 on, the Emergency Service implemented the MTS for risk classification of adult and pediatric patients, performed by nurses in the Service.

The study population consisted of patients aged less than 14 years classified by MTS in the pediatric emergency in the period from January 1st 2013 to December 31st 2013. The study included the records of patients aged under 14 years treated after the classification made from MTS classified as Immediate, Very Urgent, Urgent, Standard, Non-Urgent and not classified. It is known that some patients sought care at the pediatric emergency room more than once during the study period. Therefore, for purposes of results, the total number of visits in the pediatric Emergency Hospital classified by the MTS will be considered.

The study excluded the records of patients admitted to the pediatric emergency after the transfer of other health institutions, previously classified by the STM.

To collect data, we requested a survey to the Medical Records Service and Health Information, which contains the computerized registration data from medical records of patients classified by MTS held by a *query*. The *query* is a way to investigate computerized records in the institution and is requested by filling out a survey form the study variables. In the query was requested all the variables relevant to the study: a) age; b) sex; c) flowchart; d) discriminator; e) classification according to the MTS (Immediate, Very Urgent, Urgent, Standard, Non-Urgent and not classified); f) referring patients after medical consultation (hospital, hospitalization, death); g) place of hospitalization (Pediatric Procedures Room, Pediatric Observation Room, Pediatric Unit, Pediatric Intensive Care Unit, Neonatal Intensive Care Unit).

The survey data were organized in Microsoft Excel database and subsequently analyzed using the SPSS (*Statistical Package for Social Sciences*), version 18. All variables were categorized and expressed in absolute frequency and relative frequency.

The research project was approved by the Research Ethics Committee - CEP / Research Group and Graduate Studies - GPPG the Hospital de Clinicas de Porto Alegre, under the number 13-0397. The ethical principles in health were respected, according to Resolution No. 466 of the National Health Council of December 12th, 2012. The authors signed a Commitment Agreement for use of data from medical records.

## Results

During the period that included the research 10,921 consultations were carried out in the pediatric emergency. Among the individuals cared, male patients prevailed (54.4%) and the predominant age group was children aged between 29 days and two years (44.5%) allocated in the category of infants. According to the MTS's risk rating, the Urgent category prevailed in relation to other categories, totaling 4,762 (43.6%) of clients cared for, secondly the category Standard with 3,713 (34.0%) of clients cared for. The category Very Urgent presented 1,791 (16.4%) clients cared for, the Un-ranked category had 546 (5.0%) cared for, followed by the categories Non-Urgent and Immediate, with 65 (0.6%) and 44 (0.4 %) clients cared for, respectively.

As for the selected flowcharts for the risk classification of pediatric patients, 43 were chosen flowcharts of the 52 existing in MTS. The flowchart "worried parents": prevailed over the other, totaling 2,446 (22.4%) clients cared for. The other flowcharts identified in the consultations were Dyspnea in children with 2,097 (19.2%); diarrhea and vomiting with 1.267 (11.6%); malaise in children with 699 (6.4%); abdominal pain in children with 678 (6.2%); Asthma 568 (5.2%); unrated with 546 (5.0%); headache with 393 (3.6%); seizures with 218 (2.0%); problems with ears 218 (2.0%). The other flowcharts were grouped in the Other category, a total of 1,791 (16.4%) clients cared for.

According to the selected discriminators from the flowcharts, 105 different discriminators were found during the rating. We chose to describe the ten key discriminators. There was prevalence of the discriminator recent event, with 1,671 (15.3%) clients cared for. The other discriminators selected in attendance were hot child with 928 (8.5%); Low O2 saturation 808 (7.4%); moderate pain with 699 (6.4%); moderate signs of pain with 590 (5.4%); not fed with 579 (5.3%); Persistent vomiting of 557 (5,1%); unrated with 546 (5.0%); Febrile 480 (4.4%); productive cough with 426 (3.9%). The other discriminators were grouped in the Other category, with 3,637 (33.3%) clients cared for.

Regarding the outcome of the consultations after the risk assessment and medical consultation, 6,749 (61.8%) of clients cared for remained under observation and / or were under the health care team in the pediatric emergency. Of these, 5,362 (49.1%) were referred to the pediatric procedures room, where they received treatment for their acute health conditions, which were discharged afterwards; and 1,387 (12.7%) were admitted to the pediatric observation room. The rest of the clients cared for, which correspond to 4,161 (38.1%) were discharged home; and 11 (0.1%) did not wait for the doctor's appointment. After the risk assessment and the completion of the first procedures and health care, some patients were hospitalized in other hospital units. Of the total number of visits in the pediatric emergency, 1,136 (10.4%) required hospitalization. Of these, the largest number of admissions occurred in the Pediatric Unit with 871 (76.7%) admissions, followed by the Pediatric Oncology Unit with 120 (10.6%) admissions; Pediatric Intensive Care Unit with 67 (5.9%) admissions; Inpatient Neonatal Unit with 33 (2.9%) hospitalizations and Neonatal Intensive Care Unit with 23 (2.0%) hospitalizations. In the Other category (1.9%) other units of the hospital were grouped, in which at least one patient was referred for hospitalization, totaling 22 admissions.

## Discussion

The results of this study help fill a gap in Brazilian literature regarding the applicability of MTS in the risk classification in a pediatric emergency. The protocol can assist in the nursing practice, giving priority to children who need an immediate health service.

In this investigation, the application of the MTS indicated a prevalence of the Urgent risk category (43.6%), representing the need for evaluation of up to 60 minutes^3^. A study conducted in the Netherlands in order to identify the parent's ability to assess the severity of fever in children and the decision to seek the emergency department, found the following distribution between the categories of MTS: Immediate (2%); very urgent (44%); Urgent (34%); standard (19%); Non-Urgent (1%)^11^. In this study there was a prevalence of the standard and very urgent categories, respectively, unlike the results of this study, conducted in Brazil, in which the patients cared for classified as urgent prevailed, although the same risk classification protocol has been used in both researches. This may be associated with the organizational culture of the countries where the studies were developed, the same could be said about the different demands and health needs of the population. Another fact that can be attributed to this difference relates to the risk classification, for it can be underestimated or overestimated by the responsible professionals. Underestimating the risk classification can pose a problem to the health of patients with serious and / or high risk, considering that these may be classified as standard or non-urgent, resulting in serious consequences for their health. The overestimated risk classification provides greater safety for the patient and also the professional. At the same time, it corroborates the increase in patients with low risk of health admitted to the emergency service lowering the consume of resources that should be directed to patients with more severe health conditions^12^ ^-^ ^13^.

As for the main selected flowcharts, there was prevalence of the flowchart worried parents, according to the MTS. One can justify the prevalence of this flowchart to the singularized perception of parents about the child's health status and fear of its worsening, together with the certainty of a resolutive service ^14^. Other factors that transform emergency services as a top choice of parents to perform the service to children are: quality and resolutivity of care; accessibility; warranty and service agility; greater technological density offered by the service and medical care performed by a pediatrician^14^ ^-^ ^15^.

A study conducted in Brazil in order to know the profile of children and adolescents treated in a pediatric emergency department found that a portion of 47.4% was classified as health situations that could be resolved in primary care level, where patients had symptoms considered "light" and there is no possibility of complicating their health status^15^. It appears that despite the expansion of primary care and the implementation of the Family Health Strategy, the demand for emergency services continues to increase. It can be attributed to the difference between the way of thinking and acting by health professionals, managers and users seeking care in the emergency department without a clear understanding of what health needs constitute real situations to be resolved in these services^14^.

In a study developed in the Netherlands^16^, on the application of the MTS in a pediatric emergency, five main flowcharts selected in patient risk classification were highlighted: dyspnea in children; diarrhea and vomiting; worried parents; abdominal pain in children; eruptions. Another study^17^, also developed in the Netherlands, found the prevalence of the following flowcharts: General flowcharts; dyspnea in children; worried parents; diarrhea and vomiting; urinary problems. By comparing the results of studies conducted in the Netherlands with this study, it is evident that the flowcharts dyspnea in children, worried parents and diarrhea and vomiting appear in the three studies described. In accordance with the results found in the study^15^ held in Brazil, the main pathologies identified in patients who arrived at the pediatric emergency were respiratory diseases (56.2%); gastrointestinal disorders (16.6%) and viruses (13.1%). As these diseases have a direct relationship with main flowcharts found, there is the need for nurses responsible for risk classification to pay attention to these signs and their severity, with the aim of establishing the service and / or appropriate referral of these patients.

In relation to the discriminators, only one study was found in the literature in which the key discriminator selected in the pediatric patients risk classification in an emergency service was described. In order to evaluate the discriminative ability of MTS to identify serious bacterial infections in children with fever, it was possible to detect the following discriminators: fever; increased respiratory effort; ache; recent event; significant medical history; significant respiratory history; Low O2 saturation; and very low O2 saturation^18^. It should be noted that in this study those discriminators were directed to patients with fever at the risk classification moment, with possible progression to severe bacterial infection. This is different from the present study, in which we considered all patients classified in the pediatric emergency.

As for the outcomes of patients in the study in order to check the rates of hospitalization and identify patients with less urgent health, they found that 37% of patients were admitted to pediatric observation and in 63% of patients some kind of procedure was performed^19^. This fact was also observed in the present study, which showed the need for further care in the pediatric procedures room in half of the population treated before discharge from the hospital. With regard to hospitalization for pediatric patients classified by the MTS, a study conducted in the Netherlands, with the aim of assessing whether the flowcharts and the MTS discriminators could be used as markers to identify the risk of hospitalization for pediatric patients with signs of fever found a hospitalization rate of 23%^17^. The results found in the aforementioned study are twice the rate of hospitalization identified in this study, which was 10.4%. One can consider this difference due to the reason that the Netherlands study included patients who sought pediatric emergency service already showing signs of fever, whereas in our study we included all services performed in the pediatric emergency without the patient showing any signs or installed pathology.

Of the total number of clients cared for in the pediatric emergency during the period of this study, most patients were discharged after an observation period on the premises of the pediatric emergency. Another study^18^ ^)^ obtained similar results, which demonstrates the importance of risk classification of specific care for pediatric patients, suggesting that the classification system used is carried out by a professional with experience in the field.

In regard to the limitations of this study, these relate to the small amount of published research on the use of the Manchester Triage System in children, particularly at the national level. Most studies found in the literature were developed in European countries, featuring the clients cared for according to the reality of European health, that diverge from the health needs and problems presented by the population in the Brazilian health systems. Another point that is configured as a limitation to this study is the use of secondary data for the realization of it, which does allow control of the variables studied.

The study brings contributions to the advancement of scientific knowledge, it shows the characteristics of the services performed in the pediatric emergency, by risk classification through the Manchester Triage System and it can help nurses in planning based actions and interventions on health needs of pediatric patients, allowing the professional to direct their activities to the demands seeking health care in urgent and emergency services, adding quality and efficiency focused on the health of children.

## Conclusion

This study allowed us to characterize the services performed by risk classification through the Manchester Triage System, identifying demographics, main flowcharts, discriminators and outcomes in pediatric emergency.

This study presents relevance for nursing, considering that the nurse is the health professional designated to evaluate and classify the patient at the time of arrival at the emergency department. When it comes to the assessment and classification of a pediatric patient, it is essential that nurses show the control and knowledge necessary for the growth and development of the different stages of a child's life, just as the specific features that this age group presents. It is suggested that the nurse responsible for the risk classification to be someone who possess technical skills, clinical reasoning, qualified listening and knowledge in pediatrics for an approach based on the uniqueness and the integrity of the child. The ideal scenario is that the nurse was a specialized and active professional in the care of patients in pediatric age, because this work requires decision-making measures inherent in the role of nurses, who must act responsibly. Whereas the Manchester Triage System is not a simple handling protocol, it requires the professional allocation of their skills and abilities during the risk classification. From the results found in this study, we see the need for further investigation of the use of the Manchester Triage System in pediatric patients.

It is suggested that future research is conducted to assess and validate the applicability of this classification instrument in pediatric emergency services in Brazil, with a view to conducting research on the sensitivity and specificity of this risk classification protocol in pediatric care.

The study results showed key aspects in the process of risk classification by the Manchester Triage System, elucidating the main flowcharts and discriminators selected in the risk classification of the services performed in the pediatric emergency. This knowledge could contribute to the nursing practices, assisting nurses in improving the classification through this system.
